# Principles of Pathological Anatomy

**Published:** 1846-03

**Authors:** 


*Principles of Pathological Anatomy.* By J. HOPE, M. D., F. R. S., Physician to the St. Mary-le-Bone Infirmary, &c. &c. First American Edition, edited by L. M. LAWSON, M. D., Professor of General and Pathological Anatomy and Physiolgy in the Transylvania University. Philadelphia: LINDSAY & BLAKISTON, 1846, pp. 424.

This is a splendid and valuable work, containing no less than two hundred and sixty beautifully colored plates, illustrating almost every variety of structural change to which the different organs are liable. The descriptions are clear and brief, the print large and distinct, and the whole, in a word, presents itself in a most masterly and workmanlike manner.

